# Ratio of Deaths from Chloroform in England

**Published:** 1867-09

**Authors:** Edmund Andrews

**Affiliations:** 23 Cecil Street, Strand, London


### RATIO OF DEATHS FROM CHLOROFORM IN ENGLAND.

LONDON, July 13, 1867.

The London surgeons, with the exception of Dr. Protheroe Smith, and part of the officers of Guy's Hospital, have, with one consent, settled themselves down in the comfortable delusion that the risk of chloroform is scarcely worth considering. I find them just like Americans in one thing—they hate statistics. When I ask them what the actual risk of chloroformization is, they reply, "Oh, a very trifle, a mere nothing." The celebrated Mr. Simon, of Guy's Hospital, told me that it was in his opinion safer to take chloroform than to take a ride on a railroad. Others, when pressed for an opinion, generally fall back on Mr. Sansom's Handbook on Chloroform, which estimates the deaths from Chloroform to be only one out of every 17,000 persons taking it. This may be set down as the opinion of the mass of London surgeons, both eminent and otherwise. Now, while they are gliding along in this agreeable state of mind, I have been at work to gather facts, and I find that instead of one death in 17,000, one patient dies out of every 3,461 anesthetized, in the very hospitals where these gentlemen are at work. In other words, the deaths from chloroform under their own hands are about five times as numerous as they state, and as Mr. Sansom's Handbook estimates. If there were a railroad in London which killed one out of every 3,461 of its passengers, I imagine one would hesitate to buy a ticket on it. In examining Mr. Sansom's statistics, to see how he arrives at his surprising estimate, I find the reason of his error. He omits entirely the London hospitals, and takes no note of the fact that several deaths from chloroform occurred in the very one of which he was an officer. He obtains from some source an estimate of the number of times the article had been given in the hospitals of Birmingham, and other inland cities, amounting in all to 17,000 times. In these hospitals only one death from its use had "been reported," *ergo* chloroform causes only one death in 17,000 cases. Now, the very first step which I made in this investigation showed me that many deaths from anesthetics occur both in this country and America, which are never publicly reported. There are no regular statistics of anesthesia kept in any hospital here, that I have yet found, and as a chloroform death is an ugly, uncomfortable fact, it usually slumbers unnoticed by the records. The difficulties of my investigation, therefore, have been very great. My mode of obtaining the facts had been this. I have made careful personal inquiries of the surgeons, house surgeons, dressers, and secretaries of every hospital which I have visted, as to the two points, viz.: the number of times per annum chloroform has been given, as far back as their means of information extend, and, secondly, the number of deaths it has occasioned in the same period. These officers usually have no difficulty in estimating approximately the number of administrations per annum which have occurred for several years, and the deaths during the same periods. In this way I have collected figures from fourteen hospitals in Liverpool and London. I obtained reliable accounts of chloroform being administered eighty-three thousand fifty-nine times, (83,059,) and of these, twenty-four (24) proved fatal, or one in three thousand four hundred and sixty-one, (3,461.) Now, if this is "safer than riding on a railroad," then I shall buy no more railroad tickets. A railroad in active business which should have a mortality like this would kill from 500 to 3,000 passengers every year. I shall continue my investigations on this subject both here and in France, and will report any new facts which I may ascertain. Some of the hospitals here are a little uneasy, after all, and are using a new inhaler for safety, called Clover's apparatus, the principle of which is to inhale from a large air sack, which is inflated by a bellows, of a known capacity. The air, in passing from the bellows, goes through a hot evaporator, containing just enough chloroform by measure to give 3 or 3½ per cent. of chloroform vapor to the air in the sack. The huge, black bag, about 3 feet long and $2\frac{1}{2}$ feet wide, is slung on the back of an assistant, looking like the burden in the pictures on the back of Bunyan's Pilgrim. A large tube passes under his arm, terminated by an inhaler so arranged with valves that the patient inspires from the bag and expires into the open air. It is too clumsy for private practice, but works well in hospital, and probably promotes safety, as the patient cannot possibly get more than the per cent. of chloroform vapor which is placed in the sack. It has not been used enough yet to test, practically, the per cent. of mortality under it.

For the present, therefore, it would seem to be our duty to use ether in all cases in which the patient can be readily brought under its influence, and to reserve chloroform for those whom ether will not subdue. Possibly the tetrachloride of carbon, now being introduced by Dr. Protheroe Smith, may prove to have the safety of ether and the promptness of chloroform. If so, all good surgeons will rejoice. I should have stated that my chloroform statistics are gathered exclusively from surgical cases, and are of no value in determining the safety of the article in midwifery.

Notwithstanding the deficiency of Mr. Sansom's book, in estimating the real danger of chloroform, it will be found to contain very valuable information on other branches of the subject, together with the Report of the Committee of the Royal Medical and Chirurgical Society, in 1864, may be said to contain the best results of English investigation up to the present date.

EDMUND ANDREWS, M.D.

*23 Cecil Street, Strand, London.*

